# The role of simulation in mastering highly specialized pediatric surgery: current trends and future perspectives

**DOI:** 10.1515/iss-2025-0015

**Published:** 2025-11-05

**Authors:** Louisa Schuffert, Sabine Drossard

**Affiliations:** Klinik und Poliklinik für Kinderchirurgie, Universitätsmedizin Mainz, Mainz, Germany; Klinik und Poliklinik für Allgemein-, Viszeral-, Transplantations-, Gefäß- und Kinderchirurge, Universitätsklinikum Würzburg, Würzburg, Germany

**Keywords:** simulation based training, pediatric surgery training, pediatric surgery, surgery training, simulation

## Abstract

The acquisition of surgical skills in highly specialized pediatric surgical procedures – particularly the management of rare congenital anomalies – poses significant educational challenges. Traditional training models relying on apprenticeship and intraoperative exposure are often insufficient due to the rarity and complexity of such conditions. Simulation-based training offers a structured, reproducible, and risk-free environment to practice and refine surgical techniques. This scoping review examines the current landscape of simulation in pediatric surgery, focusing on anorectal malformations (ARM), esophageal atresia (EA), Hirschsprung’s disease (HD) and congenital diaphragmatic hernia (CDH). A dual search strategy was applied: a systematic literature search via PubMed and a structured online search via Google for commercially available simulation models. A range of models was identified across the four index conditions, including low-fidelity, high-fidelity, hybrid, and animal tissue-based simulators. EA and CDH showed the highest diversity in simulation tools, supporting both open and minimally invasive approaches. Several of the models we identified have demonstrated face, content and construct validity, but systematic data on learning outcomes and user satisfaction remain scarce. While low-fidelity models are useful for basic skill acquisition, high-fidelity and hybrid simulations more closely replicate the operative environment and enhance decision-making and technical proficiency. However, access to high-fidelity simulators remains limited due to cost and resource constraints. By identifying current limitations and opportunities of simulation training, this scoping review provides insights into how the sustainable training of the next generation of pediatric surgeons can be mastered.

## Introduction

### Challenges in training highly specialized pediatric surgery

Pediatric surgery encompasses a wide range of complex and often rare procedures, particularly in the treatment of congenital anomalies. Acquiring proficiency in highly specialized pediatric surgical techniques, such as the repair of esophageal atresia, anorectal malformations, Hirschsprung’s disease, and congenital diaphragmatic hernia, presents significant challenges. These procedures require advanced technical skills, meticulous tissue handling, and a profound understanding of intricate anatomical variations. However, their low incidence limits the trainees’s exposure, making it difficult to gain sufficient hands-on experience and raising critical questions about how these unique skills can be effectively taught and acquired in the future.

One of the primary challenges lies in the steep learning curve associated with these rare procedures. Beyond technical expertise, an in-depth comprehension of the underlying anatomy and pathology is essential, as each case can present unique anatomical variations. Furthermore, ethical concerns arise regarding direct trainee involvement in high-risk surgeries on pediatric patients, where patient safety must take precedence over educational needs. The traditional apprenticeship model, in which trainees gradually assume increasing responsibility under the supervision of senior surgeons, is increasingly constrained by these ethical considerations.

A promising solution to these challenges is the integration of simulation-based training. The European Training Requirements for Pediatric Surgery state that “Simulation/hands on courses should be incorporated in the training program” [1]. Simulation provides a structured, risk-free environment in which trainees can refine their surgical skills without compromising patient safety. Recent advancements have led to the development of various simulation modalities tailored to pediatric surgery. However, despite increasing research and publications on this subject, the integration of simulation into pediatric surgical curricula remains inconsistent, and not all models fully replicate the complexity of clinical procedures.

This scoping review examines the current state of simulation in pediatric surgery, with a focus on its application in highly specialized procedures. It explores key advancements, available training modalities, and their incorporation into existing educational frameworks. Furthermore, it highlights future directions and potential innovations to optimize training and ensure that the next generation of pediatric surgeons will be adequately prepared to provide the highest standard of care.

### Overview of simulation approaches

Each simulation modality has unique advantages and limitations, and their integration into pediatric surgical training depends on factors such as availability, cost, and the specific skills being taught. These tools can be broadly categorized into four main groups:

#### Low-fidelity simulation

A low-fidelity model is a simple, less detailed, or less realistic training model that conveys fundamental concepts or techniques but does not fully replicate the complexity or realistic haptic feedback of an actual procedure. Often referred to as “low-technology” or “low-cost” models, they are cost-effective and utilize physical, non-living materials to replicate key aspects of a surgical task while omitting certain complexities encountered in real-life scenarios. They allow learners to practice fundamental skills repeatedly, improving dexterity and technique. They are particularly useful for practicing essential technical skills, such as camera navigation, cutting, suturing, and grasping, where repeated training is necessary to develop proficiency [2]. Examples include suturing pads, knot-tying boards or laparoscopic box trainers.

#### High-fidelity simulation, inanimate models

These models use advanced materials and technology to create realistic anatomical structures. High-fidelity simulators, while effective, often come with substantial costs, limiting their accessibility, especially in resource-constrained settings. Examples include:

– Virtual reality (VR) and augmented reality (AR) simulators provide immersive environments where trainees can practice complex surgical procedures.
– 3D-printed models mimic patient-specific anatomy, offering opportunities for preoperative planning and skill refinement.
– Computer-based simulators with haptic feedback help simulate the tactile sensation of performing surgery.

#### High-fidelity simulation, animal and cadaveric models

Animal tissue models (e.g., chicken, pig or rabbit tissues) are used for practicing live-tissue handling, dissection, and suturing techniques. These simulations provide hands-on experience in anatomical dissection and the management of real tissues. Human cadaveric models offer a highly realistic environment for surgical training but are limited by availability, ethical considerations, and financial constraints. In pediatric surgery, cadaveric simulation is not in rarely employed.

#### Hybrid simulation approaches

Hybrid simulation combines different simulation techniques to enhance learning outcomes and better approximate real-life surgical scenarios. For example, a cadaveric model may be combined with VR overlays to guide a trainee through a complex procedure, or a low-fidelity task trainer can be incorporated into immersive simulation scenarios involving standardized patients. Hybrid models aim to bridge the gap between isolated technical training and real-life surgical experience by integrating multiple learning modalities.

### Validation of simulation models

Simulation offers valuable benefits for pediatric surgical training [2], 3], particularly in the context of rare and complex procedures. However, standardized evaluation is essential to assess long-term effectiveness and trainee acceptance. Validation helps ensure that simulation models are not only realistic but also educationally effective and appropriate for assessing performance. Face validity refers to the perceived realism of a simulation model, as judged by experts or trainees. Content validity evaluates whether the simulator includes all essential components of the procedure. Construct validity assesses whether the model can distinguish between users of different skill levels, e.g. by showing that experienced surgeons perform better than novices. Additional outcomes may include user satisfaction and the evidence of learning effects [4].

This scoping review examines the current state of simulation in pediatric surgery, focusing on its application in highly specialized procedures. It explores key advancements, available training modalities and highlights future directions to optimize training and ensure that the next generation of pediatric surgeons is adequately prepared to provide the highest standard of care.

## Methods

Based on expert consultation and consensus, four rare and complex pediatric surgical conditions were selected for analysis: posterior sagittal anorectoplasty (PSARP) for anorectal malformations (ARM), transanal endorectal pull-through (TERPT) for Hirschsprung’s disease (HD), surgical repair of esophageal atresia with tracheoesophageal fistula (EA/TEF), and repair of congenital diaphragmatic hernia (CDH).

**Figure 1:**
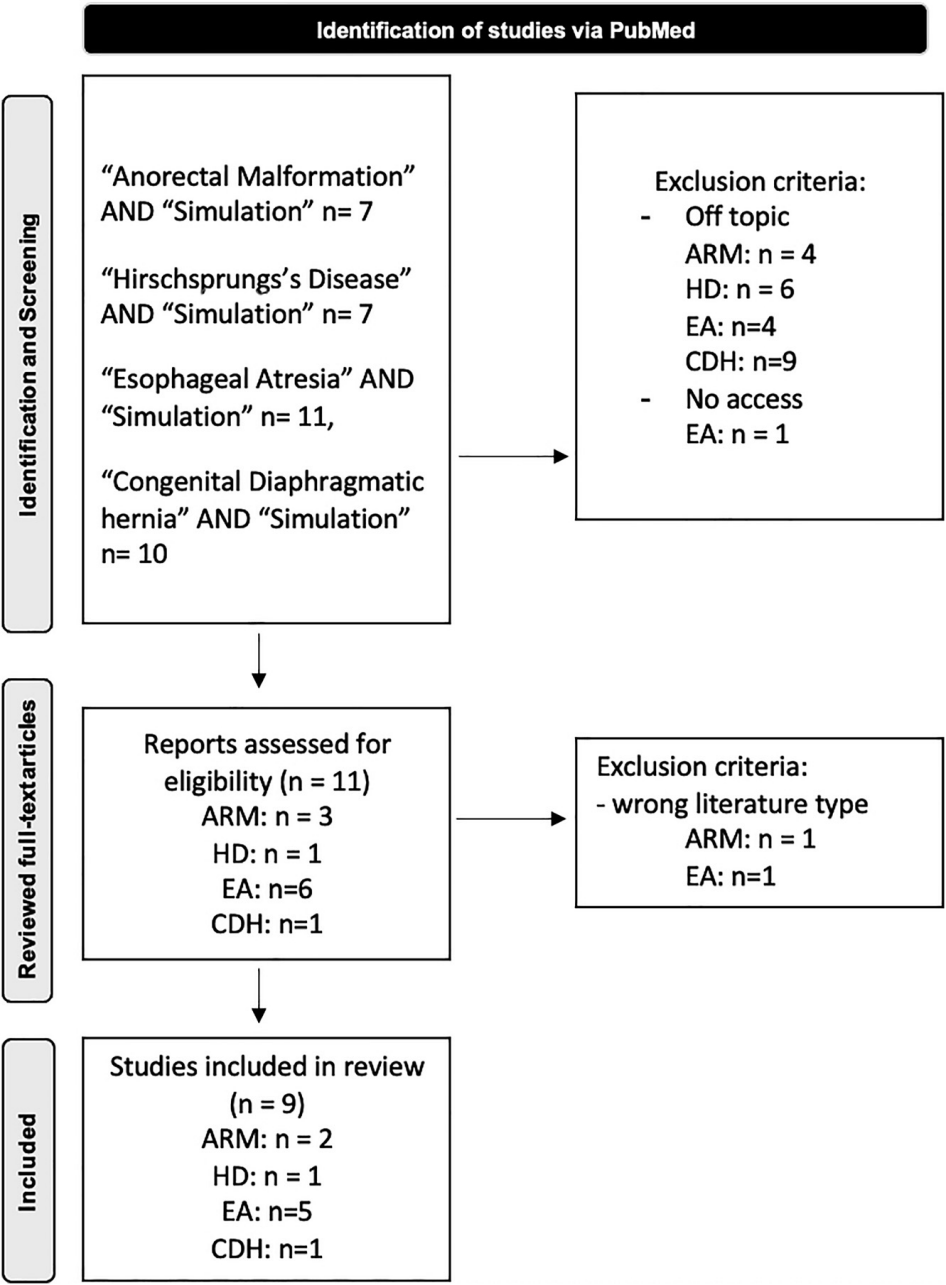
PRISMA flow diagram. ARM, Anorectal Malforamtion; HD, Hirschsprungs Disease; EA, Easophageal Atresia; CDH, Congenital Diaphragmatic Hernia.

To comprehensively assess the landscape of pediatric surgical simulation tools, we employed a two-pronged search strategy, including a systematic literature search of the PubMed database. The search strategy involved the use of controlled vocabulary terms (MeSH terms) and free-text terms. The following search terms were utilized: “Anorectal Malformation”, “Hirschsprung’s Disease”, “Esophageal Atresia”, “Congenital diaphragmatic hernia” each in combination with “Simulation”. To focus on the most recent literature, the search was filtered to only show articles published between January 2022 and February 2025. Articles were included if they were published in peer-reviewed journals, written in English and relevant to the topics Simulation in Highly Specialized Pediatric Surgery. Original research studies, review articles, guidelines, and other relevant literature types were considered. Articles were excluded if they were not related to the topics of interest, published in languages other than English or published before the year 2022. Both authors independently screened the titles and abstracts of the retrieved articles to identify relevant studies. The full-text articles were then reviewed to determine final inclusion. Discrepancies were resolved through discussion and consensus (Figure 1). Relevant information extracted from each study included authors, publication year, study design, study population, location, outcomes assessed and key findings. Validation was considered present when the study provided a clear description of the validation methodology, such as the use of structured questionnaires employing Likert scales or comparative performance assessments.

Additionally, we searched for commercially available models via Google search. Each search was performed by using “anorectal malformation”, “Hirschsprung’s disease”, “esophageal atresia”, “Congenital diaphragmatic hernia” each in combination with “Simulation Trainer”. The search was performed on March 15th, 2025.

The findings from the included studies and websites were extracted and organized in an Excel spreadsheet (Microsoft Corporation, USA) according to key findings identified across the literature to provide an overview of the current evidence on the role of Simulation in mastering highly specialized pediatric surgery.

## Results

### Simulation tools described in peer-reviewed literature

This subsection presents simulation tools identified through the PubMed search, with a focus on their application in training for specific congenital conditions. The results are shown in Table 1. For each condition, we examine which simulation modalities were used and how they were applied.

**Table 1:** Simulation tools described in peer-reviewed literature.

Condition	Simulation modality	Description	MIS	Year	Reference	Validation
Anorectal malformations	Low-fidelity	Physical model simulating posterior sagittal anorectoplasty (PSARP)	No	2022	[5]	Face, content, construct
	High-fidelity, animal	Animal tissue model (chicken cadaver), PSARP for ARM with vestibular fistula	No	2023	[6]	None
Hirschsprung’s disease	High-fidelity, animal	Animal tissue model (piglet)	No	2023	[7]	None
Esophageal atresia with tracheo-esophageal fistula	High-fidelity, animal	Animal tissue model (chicken)	No	2022	[8]	Face, content, construct, TS, LO
	High-fidelity, inanimate	3D-printed model for open EA/TEF repair	No	2022	[9]	Face, content, construct
	High-fidelity, inanimate	3D-printed model for thoracoscopic EA/TEF repair	Yes	2023	[10]	Face, content, construct, TS
	High-fidelity, inanimate	3D-printed model for thoracoscopic EA/TEF repair	Yes	2024	[11]	Face, content, TS
	High-fidelity, inanimate	3D-printed model for open EA/TEF repair	No	2024	[12]	Face, content, construct
Diaphragmatic hernia	High-fidelity, inanimate	3D-printed models for open and thoracoscopic repair	Yes	2023	[13]	None (^a^measuring of defect size)

MIS, minimally invasive surgery; Face, face validity; Content, content validity; Construct, construct validity; TS, trainee satisfaction; LO, learning outcomes.

#### Anorectal malformations: posterior sagittal anorectoplasty (PSARP)

Anorectal malformations (ARMs) are rare congenital defects requiring precise surgical correction. To address the limited exposure to these cases during training, several simulation tools have been developed. The first model was introduced in 2022, consisting of a low-cost, low-fidelity inanimate simulation tool [14]. Due to its low cost and easy reproduction, this tool can be used for at-home hands-on training of PSARP. The focus of this training model is on learning and obtaining the steps of PSARP to adopt a structured approach. However, the lack of haptic feedback of inanimate models is a major limitation. The model was validated for face, content and construct validity [6], 7]. In 2023, a low-cost, high-fidelity tissue model using a chicken cadaver was added for simulation of posterior sagittal anorectoplasty (PSARP) in ARM with vestibular fistula [8]. Besides realistic anatomy, it is reported to offer haptic properties that mimic human tissue and operative conditions, although no formal validation has been performed.

#### Hirschsprung’s disease: transanal, endorectal pull through (TERPT)

One model has been added in recent literature: a high-fidelity, animal tissue model for the training of endorectal Soave pull-through. Developed in 2023, the model utilizes a piglet specimen [12]. According to the authors, this model offers excellent realism, thereby enhancing training experience and confidence in surgical performance. However they state that “Its limitations are that the submucosal plane dissection may be more difficult to differentiate from the (full-thickness) para-rectal plane and there may be excessive cuff eversion encountered compared with real life, due to lack of tissue tone” [12]. No validation data are currently available.

#### Esophageal atresia with tracheoesophageal fistula (EA/TEF)

Esophageal atresia (EA) with tracheoesophageal fistula (TEF) is a complex congenital anomaly necessitating intricate surgical intervention. Training in esophageal atresia repair has been enhanced by various simulation models, each with distinct advantages and limitations. The “chicken-leg anastomosis” model [9] provides a highly realistic tissue experience with good face, content and construct validity at low cost but faces ethical concerns, limited reusability, and variability in tissue quality. In contrast, 3D-printed inanimate models offer reproducibility and ethical advantages. Arredondo Montero et al. developed a high-fidelity type III esophageal atresia simulator that ensures consistent training but lacks the haptic realism of live tissue [15]. Similarly, Neville et al. developed a structured, reusable model for open esophageal atresia repair, which demonstrated face, content, and construct validity. While it was considered useful by surgeons, the model does not fully replicate the complexities encountered during actual surgery [10]. To enhance skill assessment, Choi et al. integrated motion tracking into a neonatal 3D-printed thoracoscopic simulator, improving competency evaluation but requiring additional resources [11]. Focusing on thoracoscopic training, Zahradniková et al. and Youn et al. developed high-fidelity 3D-printed models to train surgeons in minimally invasive techniques [5], 13]. These models are cost-effective and reproducible but do not fully capture real surgical conditions or patient-specific variations. All models are presented with validation results; three publications report trainee satisfaction and Zahradniková et al. additionally report learning outcomes.

#### Congenital diaphragmatic hernia repair (CDH)

Congenital diaphragmatic hernia (CDH) repair is a challenging procedure due to the delicate nature of neonatal tissues and the complexity of the defect. The Pediatric Surgical Trainees Research Network (PSTRN) developed a 3D-printed simulation models for CDH repair suitable for both via laparotomy and thoracoscopically. A validation study showed that training on the models led to reliable measurement of the diaphragmatic defect by trainees regardless of surgical experience and operative approach [16]. However, face, content, and construct validity of the model were not assessed, and no data were reported regarding trainee satisfaction or learning outcomes.

### Commercially available simulation tools

This subsection details the simulation tools identified through a Google search, highlighting commercially available models designed for pediatric surgical training that may not be represented in scientific literature. Our Google search identified at least one commercially available training model for each condition. All of these are high-fidelity, non-animated models available at varying price points (Table 2). Three manufacturers were identified: PediaTrickBoxx (Netherlands, www.pediatrickboxx.com), SurgeryLabs (United Kingdom, www.surgerylabs.net) and SurgHero (Poland, www.surghero.com). The ARM Model by PediatrickBoxx [6], 7] and the Esophageal Atresia model by SurgeryLabs [6], 7] were validated for face, content and construct validity, whereas no validation data are currently available for the other commercially available simulation tools. There is an overlap between the models described in the aforementioned publications and the commercially available models.

**Table 2:** Commercially available simulation tools.

Condition	Modality	Comment	Manufacturer	Validation	Cost
Anorectal malformations: PSARP	Low fidelity		PediaTrickBoxx	Face, content, construct	95 €
	High fidelity		SurgeryLabs	N/A	900 €^a^
Hirschsprung’s disease: TERPT	Low fidelity		PediaTrickBoxx	N/A	95 €
Esophageal atresia with tracheo-esophageal fistula	High fidelity	Open/MIS	SurgeryLabs	Face, content, construct	600 €^a^
	Low fidelity	Currently not available	PediaTrickBoxx	N/A	n.n.
	Low fidelity	Open/MIS	SurgHero	N/A	123,90 €^b^
Congenital diaphragmatic hernia repair	Low fidelity	Open/MIS	PediaTrickBoxx	N/A	10 €^b^
	High fidelity	Open	SurgeryLabs	N/A	500 €^a^
	High fidelity	MIS	SurgeryLabs	N/A	400 €^a^

MIS, minimally invasive surgery, ^a^full model with 10 replaceable parts, ^b^laparoscopy box trainer and instruments sold seperatly

High-fidelity simulation models are expensive and typically require the procurement of specialized, often single-use components for each training session. For instance, the PSARP Simulator by SurgeryLabs is priced at €900 and includes 10 replacement parts; an additional set of 10 replacement components costs €400. In contrast, low-fidelity models are significantly more affordable and rely on easily replaceable, inexpensive materials. For instance, the PediaTrickBoxx trainer, uses simple air balloons as tissue analogs, which are both readily available and inexpensive. For minimally invasive surgery (MIS) training, the use of additional box trainers and laparoscopic instruments is required. Although these involve higher initial costs, they are reusable across a wide range of procedures, offering long-term value.

In addition to commercially available simulators, there are also open-access resources – including step-by-step instructions and 3D-printable models – that enable the construction of homemade simulation devices, further reducing costs and increasing accessibility.

## Discussion and future directions

The challenges of training in highly specialized pediatric surgery are multifaceted, encompassing limited case numbers, ethical considerations, steep learning curves, patient safety concerns, and the effects of regulatory constraints. The low incidence of certain cases limits trainees’ exposure, making it difficult to acquire sufficient hands-on experience and often resulting in extended training periods [17]. Addressing these issues is essential to ensure that future pediatric surgeons are well-equipped to provide the highest standard of care for their young patients.

Minimal invasive pediatric surgery has become increasingly prevalent, requiring surgeons to develop additional skills in laparoscopic, thoracoscopic and robotic-assisted procedures. However, mastering these technologies adds further complexity to training, as exposure to rare congenital cases in a minimally invasive setting is even more limited. Simulation-based training has become an invaluable tool in pediatric surgery, particularly for rare and complex congenital anomalies. By providing a controlled environment for practice, simulation can enhance surgical skills without compromising patient safety. This scoping review highlights the recent addition of diverse simulation modalities for training in congenital anomalies, reflecting current developments in the field.

### Advantages of simulation-based training

Simulation models provide a structured, risk-free environment where trainees can repeatedly practice surgical techniques without compromising patient safety. High-fidelity models, particularly 3D-printed and animal-based simulations, offer an anatomically realistic experience, allowing for skill refinement in complex procedures. These models facilitate hands-on learning and improve confidence before clinical application. Additionally, motion-tracking technologies, as introduced in some 3D-printed models, enhance objective skill assessment and competency evaluation.

Low-fidelity models, while less realistic, serve as effective introductory tools for mastering fundamental surgical steps. Their affordability and accessibility enable broader dissemination, making them particularly useful in resource-limited settings. Furthermore, hybrid approaches that integrate multiple simulation techniques offer a promising avenue for maximizing training effectiveness by balancing cost, realism, and accessibility.

### Integration of simulation into European pediatric surgical training

The European Training Requirements (ETR) for Pediatric Surgery recommend that “simulation/hands-on courses should be incorporated into the training program” [1]. Among 43 European countries, 27 have no formal mandate, 12 implement simulation locally, and Switzerland is planning integration into its new training program [18]. The United Kingdom and Ireland explicitly recognize simulation as a “learning tool,” while only France has a nationally mandated, simulation-based curriculum for pediatric surgery residents. Breaud et al. concluded that implementing mandatory simulation training for pediatric surgery trainees is feasible, effective and well received by learner and educators, still it needs a lot of resources [19]. These findings highlight the growing recognition of the value of simulation in pediatric surgical training while underscoring the need for structural and financial support to enable its broader integration across Europe.

### Limitations and challenges

Despite their advantages, simulation models also present challenges. High-fidelity models, especially those utilizing animal tissue, face ethical concerns, limited availability, and variability in tissue quality. Although cadaveric simulation offers unparalleled realism, its high cost and ethical constraints limit widespread implementation, particularly in pediatric surgery. In contrast, 3D-printed inanimate models provide reproducibility but lack the dynamic properties of live tissue, potentially impacting haptic feedback and operative realism. Since most simulation models are designed to replicate either minimally invasive or open surgery, trainees require access to multiple models to gain proficiency in both approaches.

Although this scoping review focuses on the availability and design of simulation models, validation and educational impact remain key considerations. Two systematic reviews highlight the growing but still heterogeneous evidence base for simulation-based training in pediatric surgery [3], 4]. Several of the models we identified have demonstrated face, content and construct validity, but systematic data on learning outcomes and user satisfaction remain scarce. Furthermore, despite established validity concepts, the validation remains inconsistent across studies and models.

Another challenge lies in the inconsistent integration of simulation into surgical training programs. While evidence supports the efficacy of simulation-based education, standardization across institutions remains limited. Moreover, commercial models vary significantly in cost and quality, posing barriers to universal adoption.

### Future directions

To optimize simulation-based training in pediatric surgery, future efforts should focus on developing cost-effective yet realistic models that bridge the gap between inanimate and live-tissue simulations. Innovations such as augmented reality (AR) and virtual reality (VR) may further enhance the learning experience by providing immersive, interactive environments tailored to specific surgical procedures. Additionally, integrating standardized simulation curricula into residency training could ensure consistent exposure and skill acquisition across training programs.

Ultimately, to justify investment in simulation-based training, further research is required to assess its impact on real-world surgical outcomes. Key areas for future investigation include the correlation between simulation performance and surgical proficiency, large-scale trials assessing the effectiveness of different simulation modalities in improving patient safety and surgical quality and evaluation of the economic feasibility of integrating advanced simulation technologies into training programs.

By refining simulation modalities and improving accessibility, we can better prepare the next generation of pediatric surgeons to master complex and rare surgical procedures while maintaining patient safety and surgical excellence.

## Conclusions

Simulation-based training has emerged as a critical component of pediatric surgical education, particularly for rare and complex congenital anomalies. While current models offer significant advantages, ongoing advancements are needed to enhance realism, accessibility, and integration into standardized training curricula – for open and minimal invasive surgery. For very rare congenital anomalies, simulation is not only relevant for residents but also for experienced colleagues. By embracing technological innovations, expanding global access, and conducting rigorous validation studies, the future of pediatric surgical simulation holds immense potential to improve both trainee competencies and patient outcomes.
